# Isolation of the Initial Bovine Alphaherpesvirus 1 Isolate from Yanbian, China

**DOI:** 10.3390/vetsci11080348

**Published:** 2024-08-01

**Authors:** Jingrui Hao, Jingfeng Fu, Kai Yu, Xu Gao, Keyan Zang, Haoyuan Ma, Haowen Xue, Yanhao Song, Kunru Zhu, Meng Yang, Yaning Zhang

**Affiliations:** 1 Department of Veterinary Medicine, College of Agricultural, Yanbian University, Yanji 133002, China; m17808001838@163.com (J.H.); fjf19971111@icloud.com (J.F.); 13044365811@163.com (K.Y.); bakougenn@outlook.com (H.M.); xhw704601416@foxmail.com (H.X.); syh20230516@163.com (Y.S.); zkr13596766195@163.com (K.Z.); y2439082877@outlook.com (M.Y.); zyn55082023@163.com (Y.Z.); 2 Department of Animal Disease Prevention and Control Centre, Longjing 133400, China; sk-xp@126.com

**Keywords:** bovine alphaherpesvirus 1, BoAHV1, isolation and identification, infection dynamics

## Abstract

**Simple Summary:**

Bovine infectious rhinotracheitis (IBR) is a severe, febrile, and highly contagious disease caused by bovine alphaherpesvirus 1 (BoAHV1), resulting in substantial economic losses in the cattle industry worldwide. Despite its significant impact, there is a dearth of comprehensive research on the genetic characteristics and infection dynamics of BoAHV1. This study represents a pivotal milestone as it successfully isolated a BoAHV1 strain for the first time from a cattle farm in Yanji city, revealing the genetic evolutionary characteristics of BoAHV1 and the expression dynamics of its associated glycoprotein genes within host cells. Notably, the exceptional disease resistance displayed by Yanbian yellow cattle has rendered previous reports on their susceptibility to BoAHV1 infection nonexistent. These findings underscore the importance of global collaboration in understanding and combating BoAHV1, offering crucial insights into its infection dynamics during host cell invasion.

**Abstract:**

Bovine infectious rhinotracheitis (IBR), caused by bovine alphaherpesvirus 1 (BoAHV1), poses significant challenges to the global cattle industry due to its high contagiousness and economic impact. In our study, we successfully isolated a BoAHV1 strain from suspected infected bovine nasal mucus samples in Yanji city, revealing genetic similarities with strains from Sichuan, Egypt, and the USA, while strains from Xinjiang, Beijing, Hebei, and Inner Mongolia showed more distant associations, indicating potential cross-border transmission. Additionally, our investigation of BoAHV1 infection dynamics within host cells revealed early upregulation of *gB*, which is critical for sustained infection, while the expression of *gC* and *gD* showed variations compared to previous studies. These findings enhance our understanding of BoAHV1 diversity and infection kinetics, underscoring the importance of international collaboration for effective surveillance and control strategies. Furthermore, they lay the groundwork for the development of targeted therapeutics and vaccines to mitigate the impact of IBR on the cattle industry.

## 1. Introduction

Bovine infectious rhinotracheitis (IBR), also known as red nose disease, is an acute, febrile, and contagious illness caused by bovine alphaherpesvirus 1 (BoAHV1) [1,2]. Clinically, it presents with inflammation of the respiratory tract and tracheal mucosa, dyspnea, and nasal discharge [3]. Additionally, it results in genital tract infections, conjunctivitis, meningoencephalitis, abortion, mastitis, and other ailments [4]. This disease is classified as a notifiable disease by the WOAH and a Class II animal infectious disease in China. It is influenced by viral virulence, host immune responses, host age, environmental conditions, and concurrent bacterial infections [5]. Moreover, IBR is a primary risk factor for bovine respiratory disease (BRD) [6], which is the most economically impactful disease in the cattle industry [7,8]. Concurrent infection with BoAHV1 and Mannheimia hemolytica (MH) is often linked to the development of BRD.

BoAHV1 is a member of the order Herpesvirales, family Orthoherpesviridae, subfamily Alphaherpesvirinae, genus Varicellovirus, and species Varicellovirus bovinealpha1 [9]. First identified in Los Angeles, USA, in the early 1950s [10,11], the virus was initially isolated by Madin et al. [12]. Subsequent studies recovered the virus from various tissues, including the conjunctiva, brain, vulva, and fetus of infected cattle [13]. In 1980, the first BoAHV1 strain was isolated in China from cattle imported from New Zealand [14]. Serological studies conducted across national cattle ranches revealed the presence of BoAHV1 in dairy cows, buffaloes, yellow cattle, and yaks [15,16]. BoAHV1 strains are classified into three subtypes, BoAHV1.1, BoAHV1.2a, and BoAHV1.2b, based on antigenic and genomic analyses [17]. BoAHV1.1, associated with the ‘classical’ form of IBR, primarily induces respiratory symptoms and is typically isolated from the respiratory tract and aborted fetuses [18]. Conversely, BoAHV1.2 predominantly infects the reproductive tract, leading to various reproductive disorders. As another significant species of bovine herpesvirus, BoAHV5 primarily induces diseases associated with the nervous system [19].

At the molecular level, BoAHV1 is a triple-component, double-stranded DNA (dsDNA) virus with a core, capsid, and envelope that measures approximately 150 to 200 nm in diameter [20]. The capsid takes the form of a stellate icosahedron with 162 radially arranged capsomeres enclosed within a lipid-containing membrane. The nucleocapsid, consisting of a core and a capsid, forms through the coiling of double-stranded DNA around proteins [21]. The BoAHV1 genome includes a long unique region (UL) of approximately 106 kb, a short unique region (US) of approximately 10 kb, and two identical inverted repeats (TRS and IRS), each approximately 11 kb [22]. It contains 73 open reading frames (ORFs) that encode 33 structural proteins [23,24]. Previous studies have highlighted the essential role of several glycoproteins, including *gB*, *gC*, *gD*, *gE*, *gH*, *gK*, and *gL*, derived from BoAHV1 in facilitating virus–cell interactions, with *gB* being the most conserved [25]. BoAHV1 can be transferred directly from infected cells to nearby uninfected cells by *gB*, which can cause the virus to enter target cells [26,27]. Notably, these glycoproteins adhere to receptors on the surface of host cells, mediating fusion between the virus and the cellular membrane, thereby facilitating viral entry into the intracellular environment. These pivotal processes are indispensable prerequisites for the effective invasion of host cells by the virus, constituting fundamental events in viral infection [28].

In this study, a BoAHV1 virus strain was isolated for the first time from nasal fluid samples obtained from suspected infected cattle at a cattle farm located in Yanji city. Subsequent analyses were conducted to characterize the genetic features and infection dynamics. Yanji city, located in the Yanbian Korean Autonomous Prefecture of China and bordering both North Korea and Russia, serves as a crucial hub for exchanges due to its strategic location. Yanbian yellow cattle, which are highly prized for their genetic traits resulting from extensive natural and artificial selection, are valuable assets in China’s husbandry [29]. Prior to this study, BoAHV1 had not been detected in Yanbian yellow cattle. Despite the exceptional disease resistance of Yanbian yellow cattle, the widespread dissemination of the BoAHV1 virus has significantly compromised this attribute. Therefore, a comprehensive understanding of the genetic variability and replication dynamics of BoAHV1 within host cells is crucial for developing effective control and prevention strategies.

## 2. Materials and Methods

### 2.1. Cell Line and Sample Collection

The MDBK cell line (Thermo Fisher Scientific, Waltham, MA, USA) was stored in liquid nitrogen in our laboratory. The cells were cultured in a cell culture mixture comprising 80% DMEM (Thermo Fisher Scientific, Waltham, MA, USA), 20% FBS (Tianhang, Zhejiang, China), and 1% penicillin-streptomycin. Clinical specimens were obtained from cattle suspected of having BoAHV1 infection at a farm located in Yanji city, China. The swab collection was authorized by management personnel. Mucosal samples from the nose cavity of 50 potentially infected cattle were collected using swabs. The sampling procedure was carried out with minimal disruption to the animals while the sampled cattle continued to be reared at the cattle farm. Subsequently, the collected samples underwent three freeze-thaw cycles, followed by dilution in a tenfold volume of phosphate-buffered saline (PBS) solution and homogenization using a tissue grinder. Finally, the samples were stored at −80 °C in our laboratory.

### 2.2. Cloning and Sequence Analysis of the gB Gene

The DNA extracted from the samples was obtained using a viral genomic DNA extraction kit (COWIB, Nanjing, China). Primer design for amplification of the partial *gB* gene sequence of the BoAHV1 strain (accession number: MG407792), obtained from GenBank, was conducted using Oligo6.0 software (Molecular Biology Insights, Amsterdam, USA). Primers for BoAHV1-*gB*-F/R were synthesized accordingly (Table 1). The PCR amplification process involved initial denaturation at 95 °C for 5 min, followed by 30 cycles of denaturation at 95 °C for 30 s, annealing at 59.2 °C for 30 s, and extension at 72 °C for 30 s, with a final extension step at 72 °C for 10 min.

A gel recovery kit (Omega Bio-tek, Norcross, GA, USA) was used to extract the target genes following the manufacturer’s instructions. Subsequently, the purified gene fragment was ligated into the pMD-19T vector (Takara, Kyoto, Japan) and transformed into competent Trans5α cells (TransGen Biotech Co., Ltd., Beijing, China). Monoclonal colonies of *Escherichia coli* were selected and subjected to PCR identification using the aforementioned primers and reaction conditions. Positive colonies were subsequently expanded, cultured, and sent for Sanger sequencing analysis (Kumei Biotechnology Co., Ltd., Changchun, China).

### 2.3. Virus Isolation

The homogenized samples were then centrifuged at 4000 rpm for 30 min at 4 °C. After centrifugation, the supernatant was filtered through a 0.22 μm bacterial filter for virus isolation. The filtrate was inoculated into a culture flask containing 90% confluent MDBK cells at a 5% volume, and the flask was placed in a 37 °C, 5% CO_2_ incubator. MDBK cells without virus inoculation were used as the mock infection control group. The supernatant containing the virus was collected and used to inoculate MDBK cell cultures. This process was repeated for five passages to ensure viral amplification. At each passage, the virus-infected cell cultures were subjected to three freeze-thaw cycles at −80 °C to lyse the cells and release the virus. After lysis, the supernatant was collected by centrifugation, filtered to remove cell debris, and then transferred to fresh cell cultures, allowing the virus to replicate until cytopathic effects (CPE) became evident. PCR was conducted on the viral suspension collected at each passage to monitor viral presence and amplification. After five passages, the virus solution was harvested and stored at −80 °C for subsequent experiments.

### 2.4. Virus Titration (TCID_50_)

The 5th passage of the BoAHV1-YBYJ virus solution was diluted across a gradient ranging from 10^−1^ to 10^−9^ and subsequently inoculated into 96-well cell plates, with eight replicates for each dilution. The cells were then incubated at 37 °C with 5% CO_2_ for 120 h. Following the observation of cytopathic effects (CPEs), the viral titer was determined using the Reed–Muench method.

### 2.5. Virus Growth Curve

MDBK cells were seeded into individual wells of a 48-well cell culture plate at a density of 6 × 10^4^ cells/well. The BoAHV1-YBYJ virus was diluted to an MOI of 5, which corresponds to 3 × 10^5^ virus particles per well, and subsequently used to infect the cells in a volume of 1 mL. Supernatants were collected at designated time points post-infection (0, 12, 24, 36, 48, 60, 72, 96, and 120 h), followed by TCID_50_ determination. A virus growth curve was constructed, plotting infection time on the x-axis and the logarithm of TCID_50_ values on the y-axis.

### 2.6. Indirect Immunofluorescence Assay (IFA)

MDBK cells were seeded into a 6-well plate and cultured until they reached 80% confluence. Each well was then inoculated with the 5th passage of the BoAHV1-YBYJ virus solution at an MOI of 1. After 40 h of inoculation, the MDBK cells were fixed with 4% paraformaldehyde at 37 °C for 30 min, permeabilized with 0.1% Triton X-100 at room temperature for 20 min, and then blocked with 5% BSA at 37 °C for 1 h. The cells were subsequently incubated overnight at 4 °C with 1:400 rabbit anti-BoAHV1 antibody (stored by our laboratory), followed by incubation in the dark at 37 °C for 1 h with 1:200 FITC-labeled goat anti-rabbit IgG (Affinity, Suzhou, China) as the fluorescent secondary antibody. Between each step, the cells were washed three times with PBS. Finally, an inverted fluorescence microscope (Olympus, Tokyo, Japan) was used for cell observation (10× ocular, 10× objective).

### 2.7. Transmission Electron Microscopy (TEM)

The 5th passage of the BoAHV1-YBYJ virus solution (2 mL) was concentrated using a 100 kD hollow fiber column (Rigorous, Shenzhen, China), followed by infiltration of copper mesh (GE Healthcare, Marlborough, MA, USA), fixation with glutaraldehyde, and staining with 2% phosphotungstic acid. The morphology of the virus particles was subsequently identified using transmission electron microscopy (Hitachi, Tokyo, Japan), and high-resolution images were captured with a digital camera (Olympus, Tokyo, Japan). Specifically, a Hitachi HT7700 transmission electron microscope with an accelerating voltage of 180 kV and a magnification of 30,000× was used. The electron beam current was set to 10 µA, with an exposure time of 1 s. The microscope’s resolution is 0.2 nm.

### 2.8. Analysis of the Genetic Evolution of Amino Acids

The sequencing results were sorted to identify the *gB* gene sequence and compared with the sequences of reference strains in the GenBank database (Table 2). The amino acid sequence of the *gB* gene was obtained by sequencing samples clinically confirmed as BoAHV1-positive. A genetic evolutionary tree was constructed for analysis based on published sequences in GenBank. Phylogenetic trees were generated utilizing MEGA X (Mega Limited, Auckland, New Zealand), with sequence alignment performed using the ClustalW algorithm, which employs the neighbor-joining method, and analyses conducted with 1000 bootstrap replicates.

### 2.9. BoAHV1-YBYJ Glycoprotein Gene Expression in MDBK Cells

A model of infection was established using the 5th passage of the BoAHV1-YBYJ virus solution in MDBK host cells, with the uninfected group serving as the control. Negative control groups were included to ensure the success of virus infection and to rule out experimental errors and chance results. MDBK cells were seeded in 6 well plates, and each well was inoculated with the 5th passage of the BoAHV1-YBYJ virus solution at a titer of 10^5.80^ TCID_50_/0.1 mL with an MOI of 1. After 30 min of virus adsorption, the original virus solution was retained, and a cell maintenance medium was added to continue cultivation. Samples were collected from fifth-generation BoAHV1-infected MDBK cells at various time points (0.5, 1, 2, 4, 6, 8, 12, 24, 36, 48, and 60 h), and total RNA was extracted. RNA extraction was performed using a viral genomic RNA extraction kit (COWIB, Nanjing, China), with quality control measures in place to ensure the suitability of the collected RNA for subsequent experiments. The extracted RNA was then reverse-transcribed into cDNA using a reverse transcription kit (TaKaRa, Dalian, China) and stored at −80 °C. Subsequently, fluorescent quantitative PCR was performed to assess the mRNA levels of relevant glycoprotein genes at each time point. The primers used for the amplification of the *gB*, *gC*, *gD*, *gE*, *gH*, *gK*, and *gL* gene sequences of the BoAHV1 strain (accession number: MG407792) were designed using Oligo6.0 software (Molecular Biology Insights, Amsterdam, NY, USA). GAPDH was chosen as the housekeeping gene for this study. Fluorescent quantitative PCR primers for BoAHV1 were synthesized accordingly (Table 1). Quality control measures, including the assessment of reverse transcription efficiency and PCR specificity, were implemented to ensure the validity of the transcription and PCR results. The relative expression levels of target genes were determined using the 2^−ΔΔCt^ method, allowing for the comparison of gene expression changes across experimental conditions. The experimental results were analyzed using ImageJ software (NIH, Bethesda, MD, USA). One-way ANOVA was performed with GraphPad Prism software 7.00 (GraphPad Software Inc., San Diego, CA, USA). The data were subjected to normality and log-normality tests, followed by multiple comparison methods for significance testing. In our analysis, **** *p* < 0.0001, *** *p* < 0.001 and ** *p* < 0.01 indicate highly significant differences, while * *p* < 0.05 indicates significant differences. Each experiment was conducted with three biological replicates (*n* = 3) to ensure experimental reproducibility and reliability.

## 3. Results

### 3.1. PCR Analysis of Test Samples

**Figure 1:**
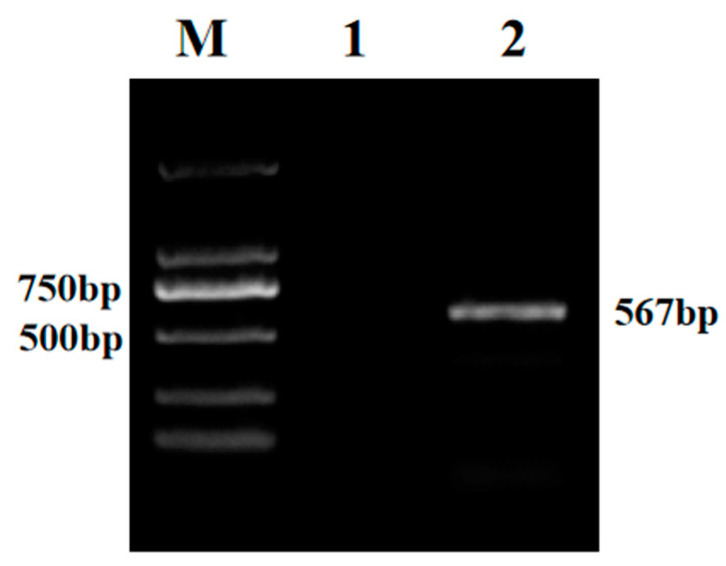
PCR detection results for pending samples. Lane M, DL 2000 Marker; lane 1, negative control; lane 2, positive sample.

Fifty samples were tested using BoAHV1-specific primers targeting the *gB* gene, revealing the presence of a specific 567 bp DNA band in seven samples, which is indicative of BoAHV1 positivity (Figure 1). In this study, the initial agarose gel data from triplicate biological replicates have been uploaded as Supplementary Material (Figure S1).

### 3.2. Virus Isolation

**Figure 2:**
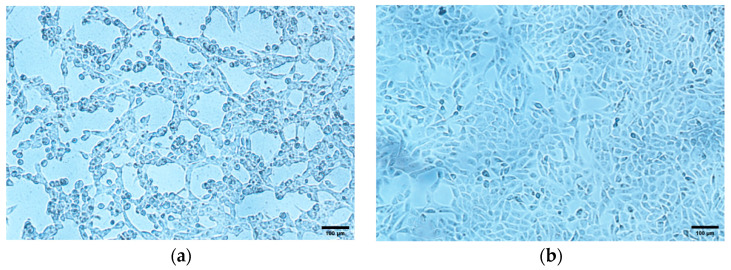

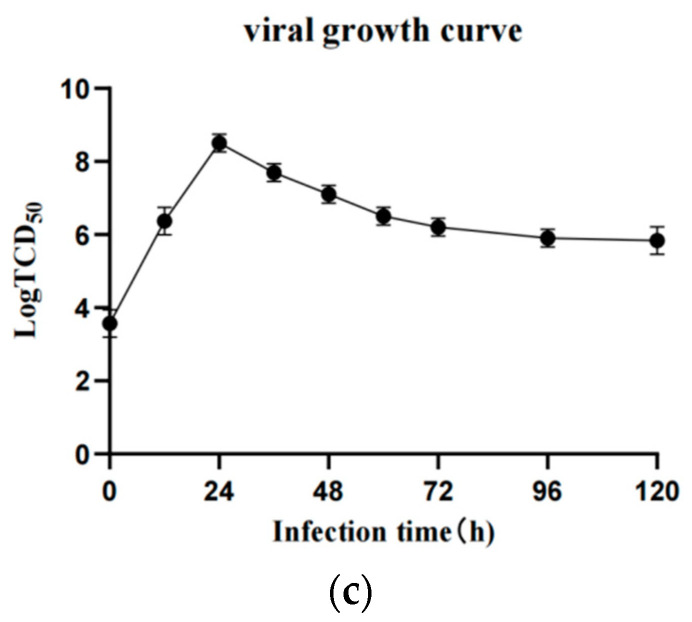
BoAHV1-YBYJ isolation from Yanbian yellow cattle. MDBK cells were infected by the 5th passage of the virus samples. CPE could be visualized 40 h post-infection (100× magnification). (**a**) Cells infected with the 5th passage of the virus sample. (**b**) Mock infection control. (**c**) BoAHV1-YBYJ virus growth curve.

Among the fifty tested samples, one of the seven BoAHV1-YBYJ positive samples identified by PCR was selected for virus isolation. In cells infected with the 5th passage inoculum, the cytopathic effect (CPE) became evident 40 h post-infection. Microscopic examination revealed cell rounding, netting, and grape-like string appearances (Figure 2a). In contrast, uninoculated cells cultured under the same conditions did not exhibit any CPEs (Figure 2b). To quantify the viral titer, the TCID_50_ of the isolated strain was determined to be 10^5.80^ per 0.1 mL (Table S1). Furthermore, viral growth kinetics indicated a significant exponential increase in viral titers within the initial phase of infection, reaching peak levels at 24 h. Subsequently, viral titers gradually declined, stabilizing thereafter by 72 h post-infection (Figure 2c).

### 3.3. Transmission Electron Microscopy (TEM)

**Figure 3:**
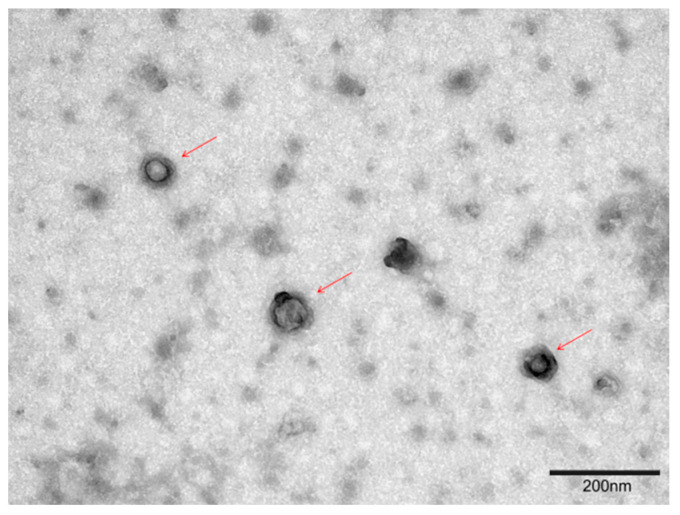
TEM was used to observe the viral morphology of BoAHV1 (30,000× magnification). Viral particles are highlighted with red arrows.

The 5th passage concentrated viral solution was observed under a transmission electron microscope. TEM revealed spherical virus particles measuring approximately 150–220 nm in diameter, each with an envelope and a capsid (Figure 3).

### 3.4. Indirect Immunofluorescence Assay (IFA)

**Figure 4:**
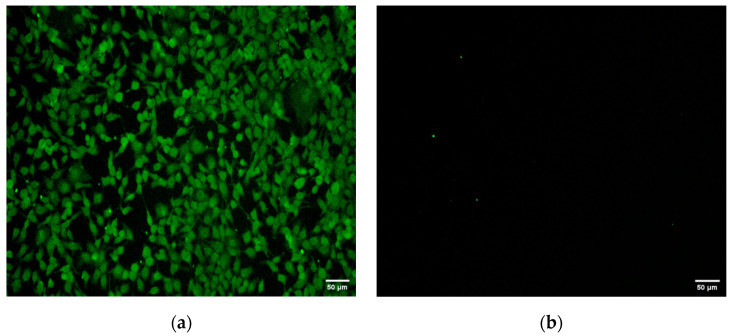
MDBK cells infected with the BoAHV1-YBYJ strain were detected using IFA. Inoculation group: distinct green fluorescence (100× magnification). (**a**) Cells infected with the BoAHV1-YBYJ strain; (**b**) mock infection control.

Immunofluorescence was used to confirm the infection of MDBK cells by the BoAHV1-YBYJ strain. In contrast to the absence of fluorescence in the control group, distinct green fluorescence was observed in the inoculated group (Figure 4), further supporting the successful infection of MDBK cells by BoAHV1-YBYJ.

### 3.5. Phylogenetic Analysis of Amino Acid Evolution in Isolates

**Figure 5:**
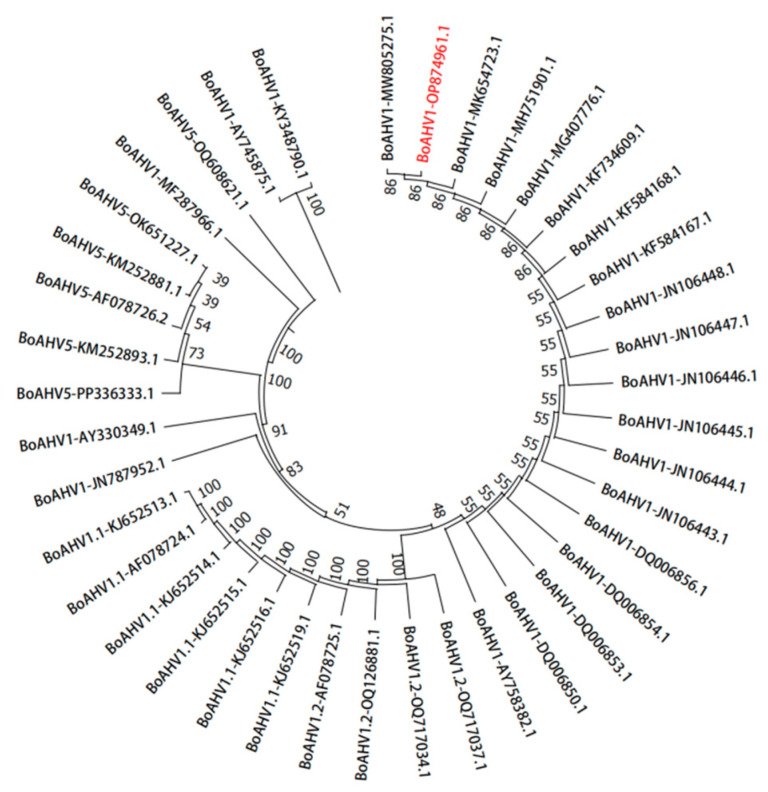
A phylogenetic tree based on the partial *gB* sequences of BoAHV1 was constructed via the neighbor-joining method. Amino acid sequences were analyzed using MegAlign software 7.1.0 (44) with a bootstrap test of 1000 replicates. The red font represents the currently isolated BoAHV1 strain (YBYJ).

The nucleotide sequence of the BoAHV1-YBYJ *gB* gene obtained from sequencing was translated into an amino acid sequence. Subsequently, 40 strains of BoAHV1 retrieved from GenBank were selected for constructing a phylogenetic tree (Figure 5). The analysis revealed an amino acid genetic evolutionary tree of the viral proteins, showing two main branches. The BoAHV1-YBYJ isolate (GenBank: OP874961) fell within the secondary branch, showing a close phylogenetic relationship with strains originating from Sichuan (GenBank: MK654723), Egypt (GenBank: MW805275), and the USA (GenBank: MH751901). Conversely, it has a more distant relationship with strains from Xinjiang (GenBank: OQ717037), Beijing (GenBank: JN106443), Hebei (GenBank: MF287966), and Inner Mongolia (GenBank: JN787952), indicating potential cross-border transmission. Furthermore, the amino acid sequence of the DNA virus BoAHV1 is highly conserved, suggesting a low probability of mutation within this virus.

### 3.6. BoAHV1-YBYJ Glycoprotein Gene Expression in MDBK Cells

**Figure 6:**
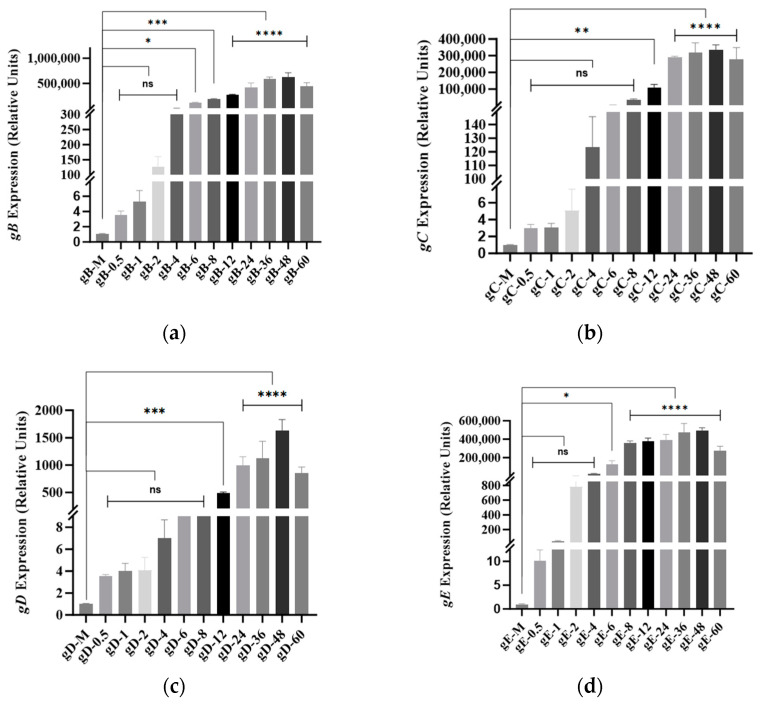

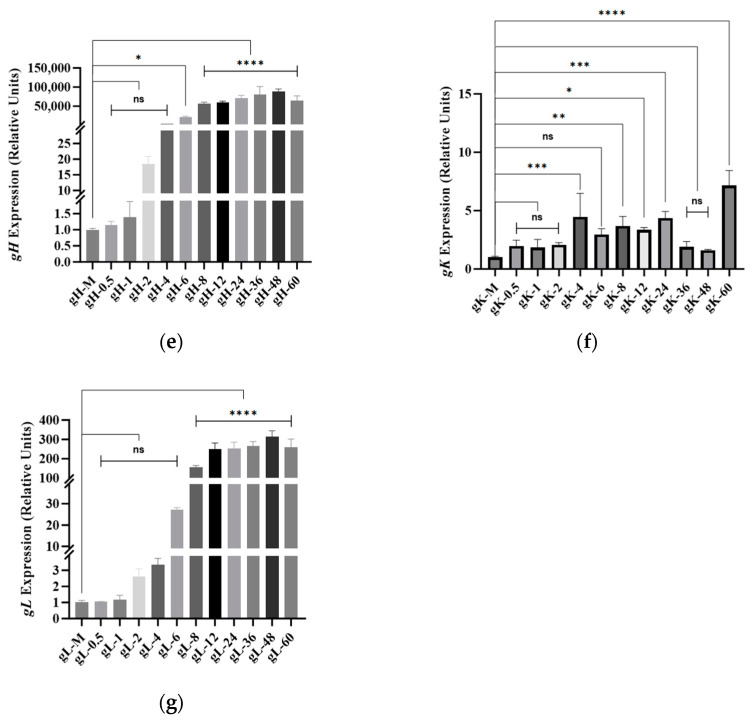
The expression patterns of BoAHV1-associated glycoprotein genes in MDBK cells over time compared with those in the mock group. (**a**) Relative *gB* mRNA expression; (**b**) relative *gC* mRNA expression; (**c**) relative *gD* mRNA expression; (**d**) relative *gE* mRNA expression; (**e**) relative *gH* mRNA expression; (**f**) relative *gK* mRNA expression; (**g**) relative g*L* mRNA expression. **** *p* < 0.0001, *** *p* < 0.001, ** *p* < 0.01, * *p* < 0.05.

After MDBK cells were infected with BoAHV1-YBYJ, GAPDH was chosen as the reference gene to evaluate the relative mRNA expression levels of various glycoproteins at different time points. Specifically, compared with those in the negative control group, the expression of the glycoprotein genes *gB* (Figure 6a), *gE* (Figure 6d), and *gH* (Figure 6e) began at 2 h post-infection, with significant upregulation evident at 6 h. Notably, *gB* consistently demonstrated the highest expression levels, emphasizing its crucial role throughout the BoAHV1 lifecycle and its potential therapeutic implications. Subsequently, at 4 and 6 h post-infection, the expression of *gC* (Figure 6b) and *gL* (Figure 6g), respectively, was detected. *gC* was significantly upregulated by 12 h, and both *gC* and *gL* peaked at 48 h post-infection. Moreover, the expression of *gD* (Figure 6c) was detected at 12 h post-infection, with relatively subdued levels observed for both *gD* and *gL* throughout the entire process. The observed infection dynamics of *gC* and *gD* in our study deviate from previous findings [30,31]. Furthermore, *gK* (Figure 6f) was minimal throughout the infection course. Additionally, the expression levels of glycoprotein genes associated with BoAHV1 in MDBK cells peaked at 48 h post-infection, indicating a subsequent reduction in gene expression. The initial triplicate biological replicate data for this study have been uploaded as Supplementary Material.

## 4. Discussion

The increasing trade of live cattle and their products, both domestically and internationally, heightens the risk of importing pathogenic microorganisms. In China, IBR is recognized as an infectious disease originating from foreign sources. The initial strain of BoAHV1 was detected in cows imported from New Zealand during the 1980s [14,15,16]. Subsequent serological surveys conducted across various cattle farms nationwide revealed the presence of BoAHV1 in dairy cows, water buffaloes, yellow cattle, and yaks. In recent years, there has been a surge in reports of BoAHV1 infections worldwide, spanning regions such as Iran [32], Brazil [33,34,35], Ireland [36], Turkey [37], Russia [38], and Italy [39]. These infections have led to significant economic losses in husbandry.

IBR extends to sheep and goats [40], while captive Asian elephants exhibit antibodies against this virus [41]. Furthermore, BoAHV1 has been isolated from asymptomatic hosts, including pronghorn antelopes, wildebeests, minks, and ferrets [42], indicating its expanding host range. Given its widespread prevalence in husbandry, it is crucial to prioritize global-scale research on BoAHV1 detection and isolation. This is essential for establishing a strong theoretical framework for mitigating its pervasive impact.

Yanbian Prefecture, comprising eight counties and cities, stands out as a key hub for China’s yellow cattle industry. As one of China’s top five local breeds, Yanbian yellow cattle play a pivotal role in the nation’s husbandry and international trade. The robust disease resistance observed in Yanbian yellow cattle is a result of various factors, such as their adaptation to natural environments, genetic predispositions, effective breeding management practices, and resilience to stressors. Consequently, no previous reports have documented BoAHV1 infection in this breed. This study isolated BoAHV1-positive cases from a cattle farm in Yanji city for the first time and successfully identified a BoAHV1 strain named BoAHV1-YBYJ. Through the analysis of the amino acid evolutionary tree of the isolated strain, we aimed to elucidate its evolutionary characteristics, thereby offering insights into the genetic diversity of BoAHV1. The BoAHV1 genes are classified into immediate early (alpha), early (beta), and late (gamma) categories based on their synthesis within infected cells. The immediate early genes of BoAHV1 (*bICP0*), early genes (*gB*, *gD*), and late genes (*gC*) have been identified in previous studies [8,43]. However, our study revealed that the expression of BoAHV1-related glycoprotein genes in MDBK cells differed from that in previous reports [30,31], primarily in terms of the expression of the *gC* and *gD* genes. Total RNA was extracted from MDBK cells infected with BoAHV1 at various time points, followed by cDNA synthesis and fluorescent quantitative PCR analysis to elucidate the temporal expression dynamics of the virus genes. These findings highlight that during viral infection, *gB* exhibits the highest expression at the mRNA level, maintaining high expression in the early stages of infection, suggesting its critical role in sustaining infection within host cells. As an essential glycoprotein, *gB* plays a crucial role in facilitating viral entry, propagation, and spread between infected cells [44]. Additionally, *gB* acts as a major antigen, inducing the host immune system to produce immune responses, including neutralizing antibodies, which makes it a key target for vaccine development [45]. Future experiments could focus on screening vaccines targeting glycoprotein *gB,* which may possess increased immunogenicity, effectively eliciting immune responses and providing enhanced protection. Moreover, drugs targeting the *gB* gene could be investigated to identify compounds capable of disrupting its function, offering promising candidates for antiviral therapeutics [46,47]. The early expression of *gC* at 4 h post-infection suggested its involvement in initial viral events, indicating a role for *gC* during the early stages of viral infection. Conversely, *gD* demonstrates a later expression compared to other genes, indicating its nonessential role in the early phases of infection. Our study findings on *gC* and *gD* gene expression dynamics diverge from those of previous reports, underscoring potential variations in viral–host interactions. At 48 h post-infection, the expression of the BoAHV1 glycoprotein peaked. The virus requires a certain duration to replicate and express its genes following cell infection, with its activity gradually waning or stabilizing in subsequent infection stages. This outcome serves as a reminder of the multifactorial regulation governing the virus replication process. It encompasses alterations in the intracellular environment of host cells and the inherent regulatory network of the virus itself. In summary, this study provides valuable insights into the dynamics of BoAHV1 infection, offering pertinent information for the development of vaccines and therapeutic strategies.

Global collaboration and sharing of BoAHV1 isolates are crucial for understanding its transmission and evolution, accelerating vaccine development and treatment strategies, and enhancing global disease control and livestock resilience against IBR. These findings provide valuable insights into BoAHV1 dynamics, paving the way for targeted interventions and vaccine development. Further exploration of the genetic and molecular characteristics of BoAHV1 strains and their interactions with host cells will aid in controlling BoAHV1 infections and safeguarding livestock health.

However, this study has certain limitations. Primarily, the sample source and scope are restricted, with a focus solely on a single yellow cattle farm in Yanbian, China. Consequently, the findings may not fully capture the virus’s spread and impact across various regions and cattle breeds. Additionally, while studies have explored the interaction between BoAHV1 and host cells, our understanding of the pathogenicity and immune evasion mechanisms of this virus in diverse hosts is limited. Therefore, future research endeavors should encompass broader sample collections from different geographic areas and host species, coupled with in-depth molecular biology and immunology analyses, to achieve a comprehensive understanding of BoAHV1 transmission dynamics and pathogenic mechanisms.

## Figures and Tables

**Table 1:** The primers used in this study.

Primers	Primer Sequences (5′-3′)	Product Size (bp)
BoAHV1-*gB*	BoAHV1-*gB*-F: GCCGTGAAGCGGAAGTT BoAHV1-*gB*-R: CCTGGTGGACAAGAAGTGG	567
BoAHV1-q*gB*	BoAHV1-q*gB*-F: GGCTCGCCAACTTCTTTCA BoAHV1-q*gB*-R: AACGGGTTCGCAATAAACG	124
BoAHV1-q*gC*	BoAHV1-q*gC*-F: CCCGTGCTGCTGTTCGTAG BoAHV1-q*gC*-R: GACTTGGTGCCCATGTCGC	176
BoAHV1-q*gD*	BoAHV1-q*gD*-F: GGATTACGAGCAAAAGAAGGTT BoAHV1-q*gD*-R: CAAAATACGGCGGAACGAC	125
BoAHV1-q*gE*	BoAHV1-q*gE*-F: GACATCCTCAACCCCTTCG BoAHV1-q*gE*-R: CTGTCGTCATCCGCAAAAG	125
BoAHV1-q*gH*	BoAHV1-q*gH*-F: CCTACTGCGGCAGCGTGTT BoAHV1-q*gH*-R: GAGGCGAGGGTTGAAGACG	137
BoAHV1-q*gK*	BoAHV1-q*gK*-F: CGCTTGCTGTCAACTTCCG BoAHV1-q*gK*-R: AACCCACGCCCAGATTTTC	188
BoAHV1-q*gL*	BoAHV1-q*gL*-F: GGCAACTTATTGCTCGCAGAC BoAHV1-q*gL*-R: GGCAAGCACCCGCCTTATA	189
GAPDH	GAPDH-F: GACCTGCCGCCTGGAGAA GAPDH-R: GAAGAGTGAGTGTCGCTGTTGA	144

**Table 2:** The sequences used in this study.

Sequence Name	GenBank ID	Location
BoAHV1	OP874961	Yanji
BoAHV1	AY330349	Brazil
BoAHV1	AY745875	Brazil
BoAHV1	AY758382	Brazil
BoAHV1	DQ006850	Brasil
BoAHV1	DQ006853	Brasil
BoAHV1	DQ006854	Brasil
BoAHV1	DQ006856	Brasil
BoAHV1	JN787952	Inner Mongolia
BoAHV1	KF584167	Israel
BoAHV1	KF584168	Israel
BoAHV1	KF734609	India
BoAHV1	KY348790	Xinjiang
BoAHV1	MG407776	USA
BoAHV1	MH751901	USA
BoAHV1	MK654723	Sichuan
BoAHV1	MW805275	Egypt
BoAHV1	JN106443	Beijing
BoAHV1	JN106444	Beijing
BoAHV1	JN106445	Beijing
BoAHV1	JN106446	Beijing
BoAHV1	JN106447	Beijing
BoAHV1	JN106448	Beijing
BoAHV1	MF287966	Hebei
BoAHV1.1	AF078724	Sweden
BoAHV1.1	KJ652513	USA
BoAHV1.1	KJ652514	USA
BoAHV1.1	KJ652515	USA
BoAHV1.1	KJ652516	USA
BoAHV1.1	KJ652519	Egypt
BoAHV1.2	AF078725	Sweden
BoAHV1.2	OQ126881	Sichuan
BoAHV1.2	OQ717034	Sichuan
BoAHV1.2	OQ717037	Xinjiang
BoAHV5	AF078726	Switzerland
BoAHV5	KM252881	Brazil
BoAHV5	KM252893	Brazil
BoAHV5	OK651227	Russia
BoAHV5	OQ608621	India
BoAHV5	PP336333	Turkey

## Data Availability

The data that support the findings of this study are available in the Supplementary Material for this article. The partial *gB* genome sequences of the BoAHV1 isolates (accession number: OP874961) have been submitted to GenBank.

## Supplementary Materials

The following supporting information can be downloaded at https://www.mdpi.com/article/10.3390/vetsci11080348/s1.
