# Supplementary material for: Isolation of the Initial Bovine Alphaherpesvirus 1 Isolate from Yanbian, China
Source: Vet Sci. 2024 Aug 1;11(8):348. doi: 10.3390/vetsci11080348 (PMC11360619; doi:10.3390/vetsci11080348)
Supplement: Supplementary file 1 [file vetsci-11-00348-s001.zip › Figure S1.pdf]

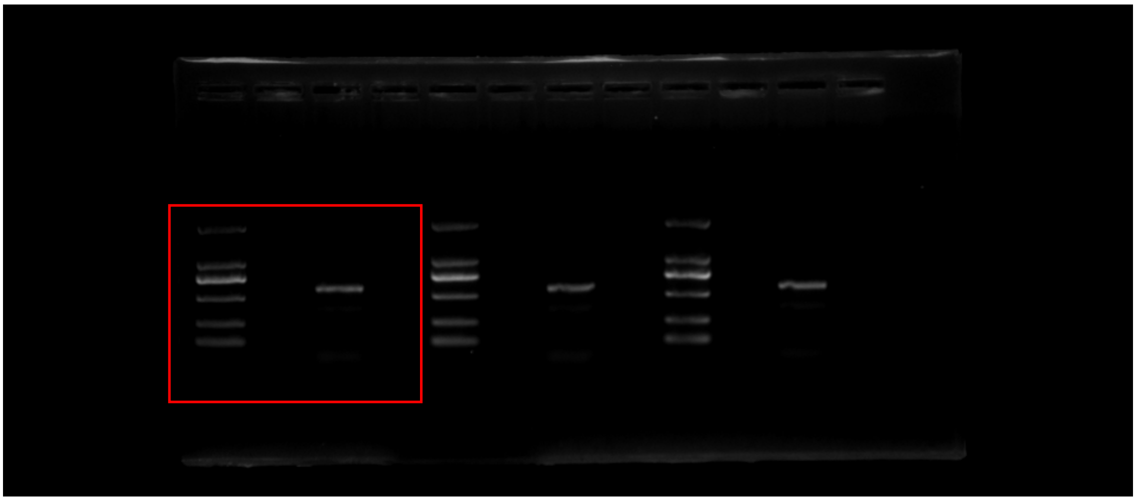

Figure S1. Agarose gel electrophoresis raw data

After harvesting purulent swab samples from the ocular, nasal, and oral cavities of the cow, genomic DNA was extracted. This was succeeded by PCR amplification and subsequent electrophoresis on a 1% agarose gel (Figure S1). A distinct 567 bp band, in accordance with the expected size, was visibly discerned. Following gel extraction and purification procedures, sequencing validation corroborated the anticipated results. To enhance clarity, one of the triplicate datasets was selectively employed, showcasing the comprehensive marker alongside the segment of the gel containing the target band, thus effectively illustrating the PCR outcome.
