# Supplementary material for: Isolation of the Initial Bovine Alphaherpesvirus 1 Isolate from Yanbian, China
Source: Vet Sci. 2024 Aug 1;11(8):348. doi: 10.3390/vetsci11080348 (PMC11360619; doi:10.3390/vetsci11080348)
Supplement: Supplementary file 1 [file vetsci-11-00348-s001.zip › Table S1.pdf]

Table S1. Determining the $TCID_{50}$ of BoAHV1 in MDBK cell culture.

Virus dilution multiples	The number of CPE holes in the same dilution ratio		Total number of CPE holes	Total number of CPE-free holes	Total number of cell pores	The percentage of CPE pores in total cell pores was calculated（%）
	Number of CPE holes	Number of CPE-free holes				
10^{-1}	8	0	42	0	42	100（42/42）
10^{-2}	8	0	34	0	34	100（34/34）
10^{-3}	7	1	26	1	27	96.3（26/27）
10^{-4}	7	1	19	2	21	90.5（19/21）
10^{-5}	6	2	12	4	16	75（12/16）
10^{-6}	4	4	6	8	14	42.9（6/14）
10^{-7}	2	6	2	14	16	12.5（2/16）
10^{-8}	0	8	0	22	22	0（0/22）
10^{-9}	0	8	0	30	30	0（0/30）
