# Supplementary figures and images for: Isolation of the Initial Bovine Alphaherpesvirus 1 Isolate from Yanbian, China
Source: Vet Sci. 2024 Aug 1;11(8):348. doi: 10.3390/vetsci11080348 (PMC11360619; doi:10.3390/vetsci11080348)

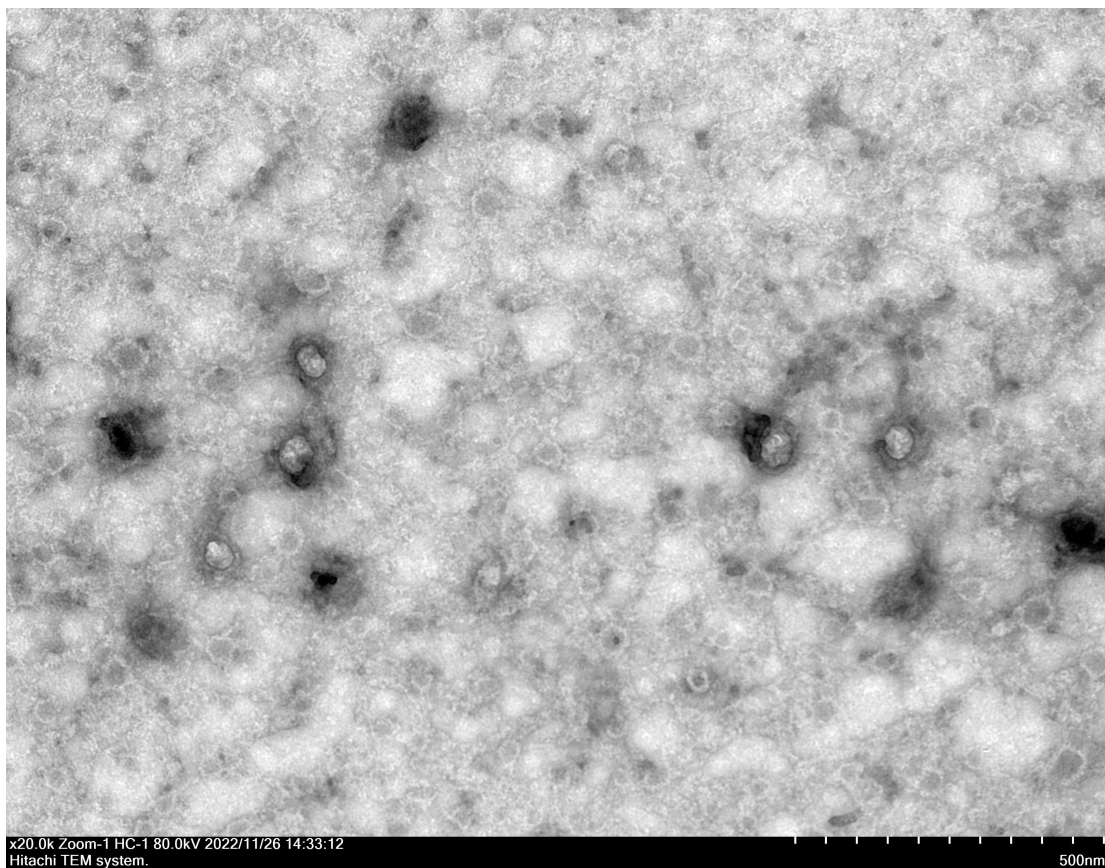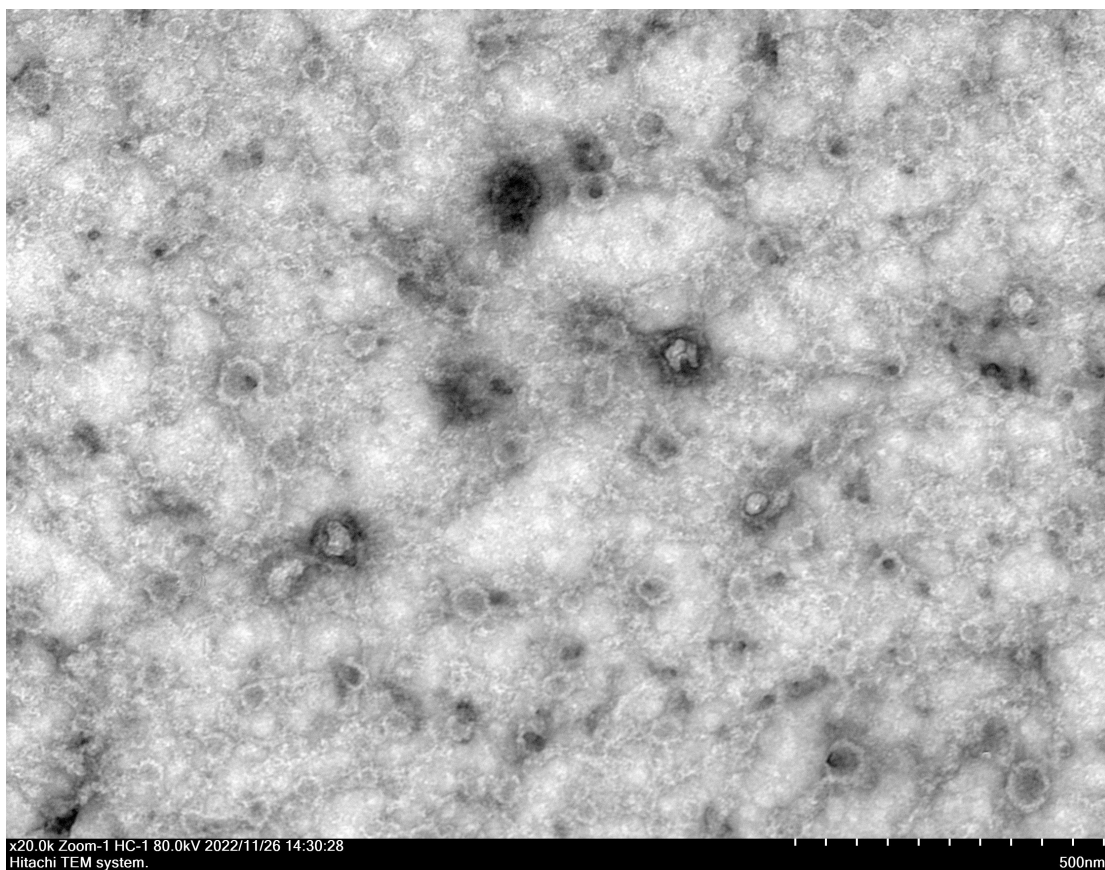

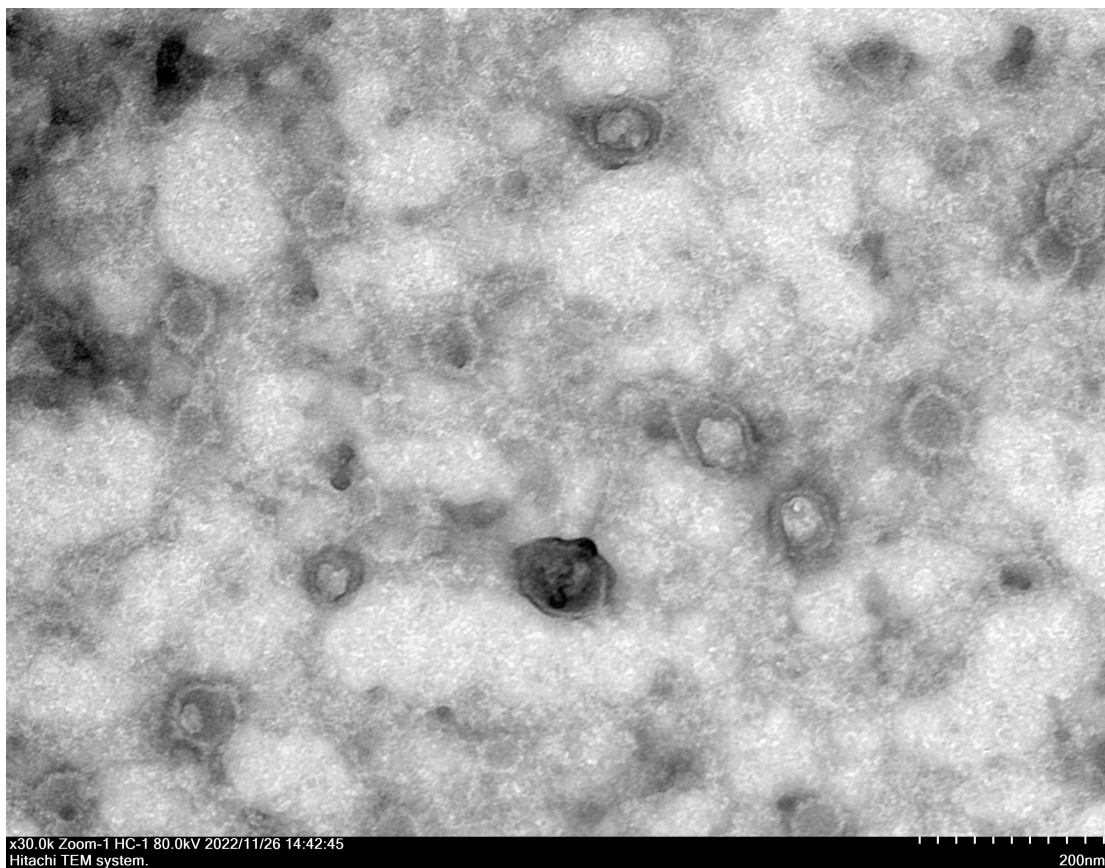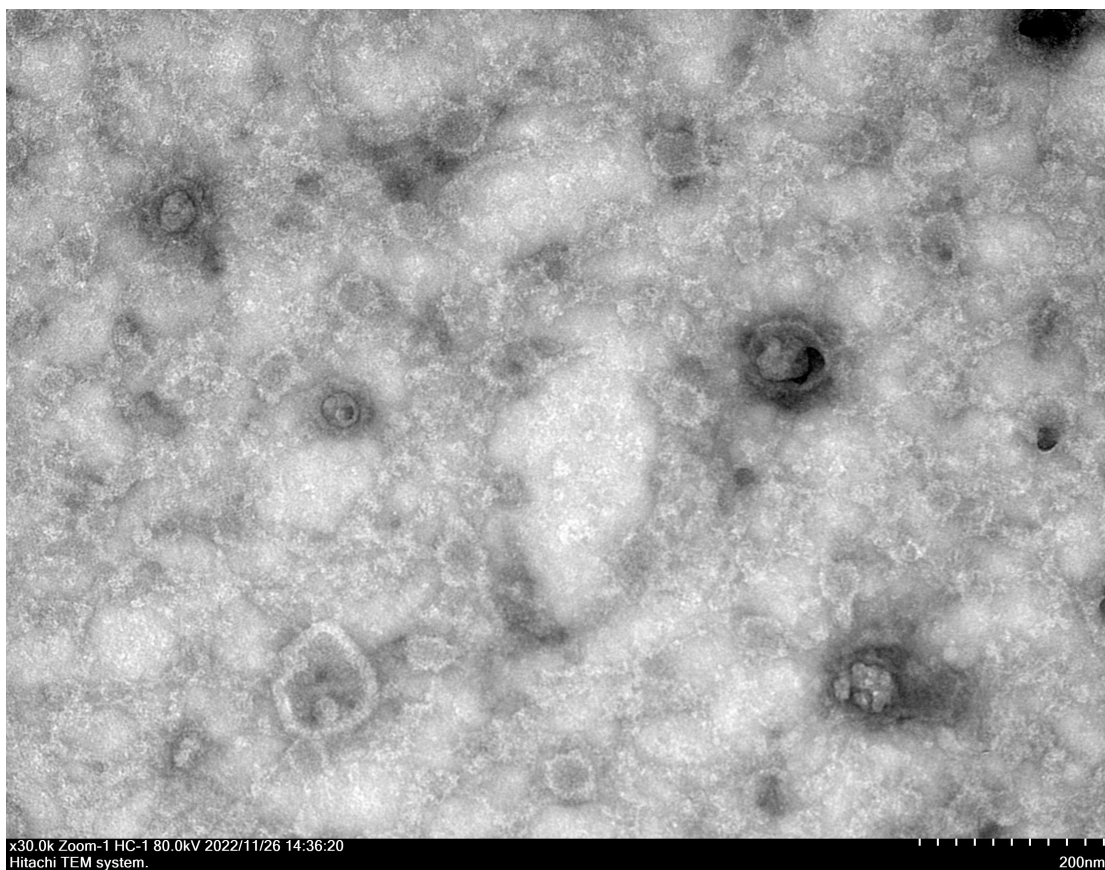

**Additional TEM figures of BoAHV1**

Supplement: Supplementary file 1 [file vetsci-11-00348-s001.zip › Additional TEM figures of BoAHV1.pdf]

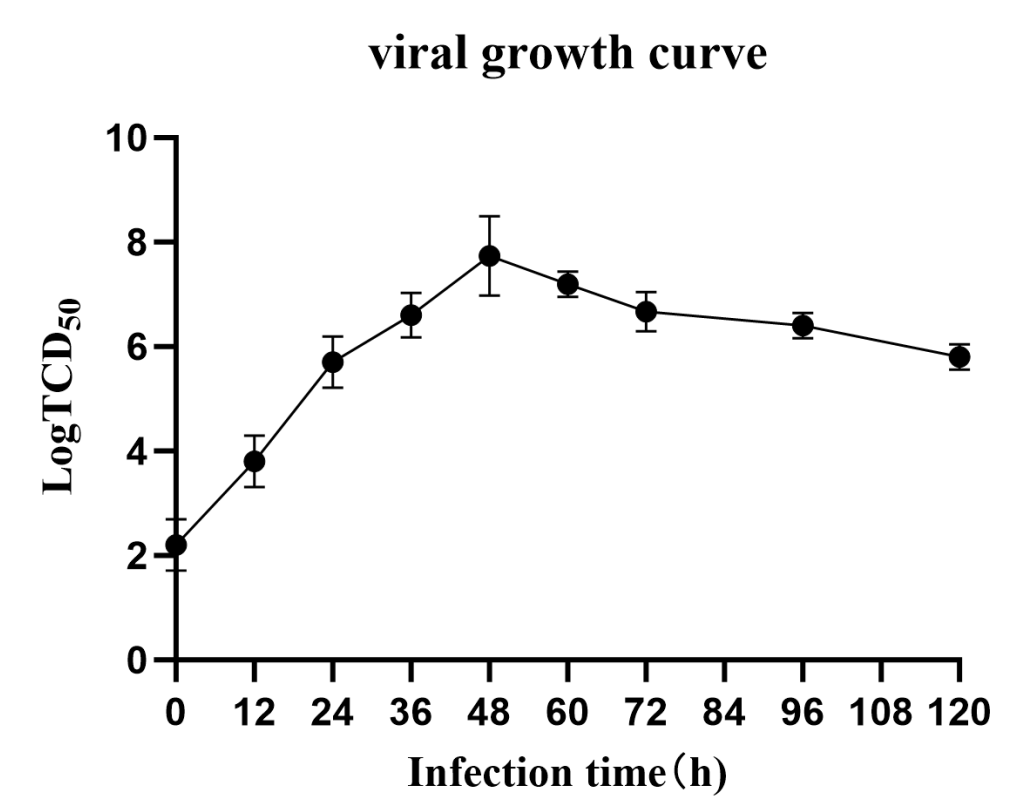

Supplement: Supplementary file 1 [file vetsci-11-00348-s001.zip › Figure S2.png]

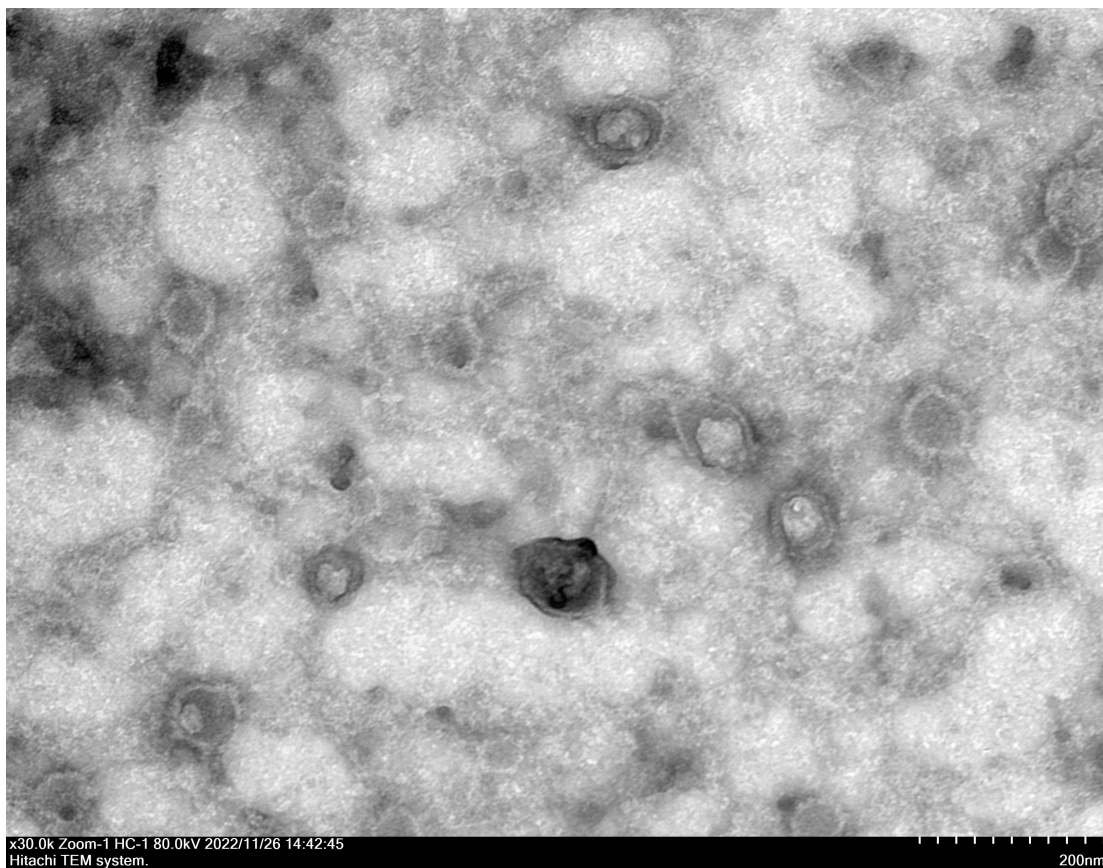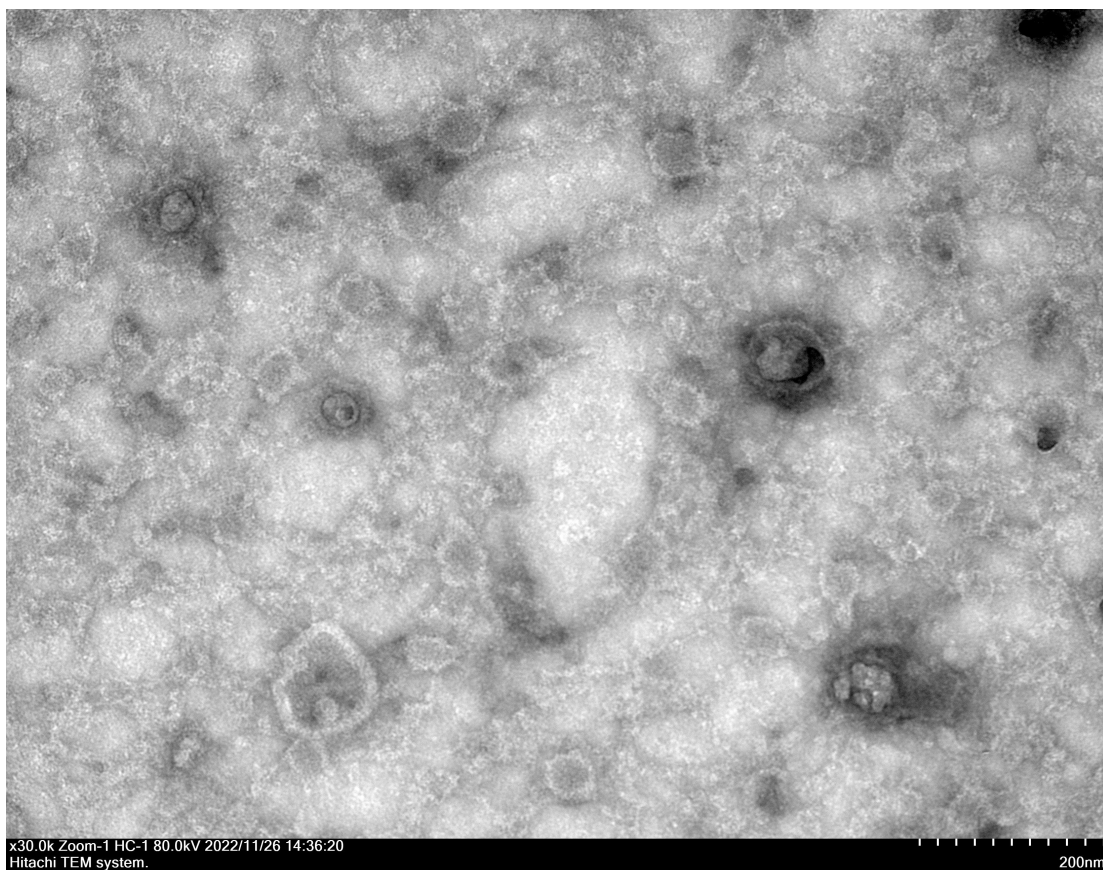

**Additional TEM figures of BoAHV1**

Supplement: Supplementary file 1 [file vetsci-11-00348-s001.zip › Supplementary figures.pdf]
